# A case of extreme weight loss due to mesenteric ischemia and antiphospholipid syndrome

**DOI:** 10.1002/ccr3.1524

**Published:** 2018-04-14

**Authors:** Nikolaos Melas, Amil Haji Younes, Robert Lindberg, Peter Magnusson

**Affiliations:** ^1^ Centre for Research and Development Uppsala University/Region Gävleborg Gävle SE‐ 801 87 Sweden; ^2^ Cardiology Research Unit Department of Medicine Karolinska Institutet Stockholm SE‐171 76 Sweden

**Keywords:** Abdominal angina, abdominal pain, antiphospholipid syndrome, mesenteric ischemia, weight loss

## Abstract

Mesenteric ischemia and antiphospholipid syndrome is a rare combination but should be suspected as a differential diagnosis. This may be presented as diffuse abdominal pain typically after food intake, diarrhea, and weight loss. Early recognition is warranted, and nutrition, stenting, and anticoagulant treatments are indicated.

## Introduction

Mesenteric ischemia (MI) accounts for 0.09–0.20% of all acute hospital admissions and represents an unusual cause of abdominal pain 1. Mortality rate is high, ranging from 24% to 94% 2.

The antiphospholipid syndrome (APS) is an acquired autoimmune disorder characterized by the presence of circulating antiphospholipid antibodies in patients with arterial or venous thromboembolism or fetal loss 3. APS can be divided into primary (53%) and secondary (47%), which is often due to systemic lupus erythematosus or other autoimmune diseases (40%) but also to infections, drugs, and malignancies (solid tumors and hematological) 4. MI due to APS is rare and accounts for no more than 1.5% of all forms of APS manifestations 5.

## Case History

A 57‐year‐old Caucasian female was referred to the gastroenterology unit because of extreme weight loss due to abdominal discomfort, fear of eating, postprandial pain, and diarrhea. She began smoking at an age of 17 years and smoked eight cigarettes daily for 35 years but reduced to two cigarettes daily the last 2 years. Her long‐term sick leave was due to back pain and depression. At childhood, she was diagnosed with epilepsy and treated with valproate sodium 2.5 g daily, which was her only medication for many years. There was no history of cerebrovascular disease among first‐degree relatives, hypertension, or diabetes mellitus. Her child was delivered after an uncomplicated pregnancy, and she had no known miscarriage. Almost 25 years ago, she underwent cholecystectomy.

Before the gastroenterology referral, she had been evaluated in primary care due to a dull epigastric pain related to food intake, typically starting half an hour after a meal and continued for an hour. Drinking provoked fewer symptoms than solid food. In primary care, she was prescribed the proton pump inhibitor omeprazole 20 mg. During the following months, her general health deteriorated and she suffered from diffuse postprandial abdominal pain, postprandial diarrhea, and weight loss. There was neither blood nor mucus in the stool. Proctoscopy at the healthcare center was normal. Laboratory markers such as C‐reactive protein, f‐calprotectin, and transglutaminase antibodies were within normal range. Next, she was referred to esophagogastroduodenoscopy at the endoscopy unit and computed tomography (CT) colonography which both were normal. The patient's symptoms were now perceived as irritable bowel syndrome. She had no contact with the primary care unit over the next half year until the same symptomatology recurred and worsened. She underwent another colonoscopy including biopsies that was normal. Unfortunately, she gradually worsened in her symptoms with significant weight loss, from 89 to 43 kg (body mass index decreased from 31.2 to 15.1 kg/m^2^).

**Figure 1:**
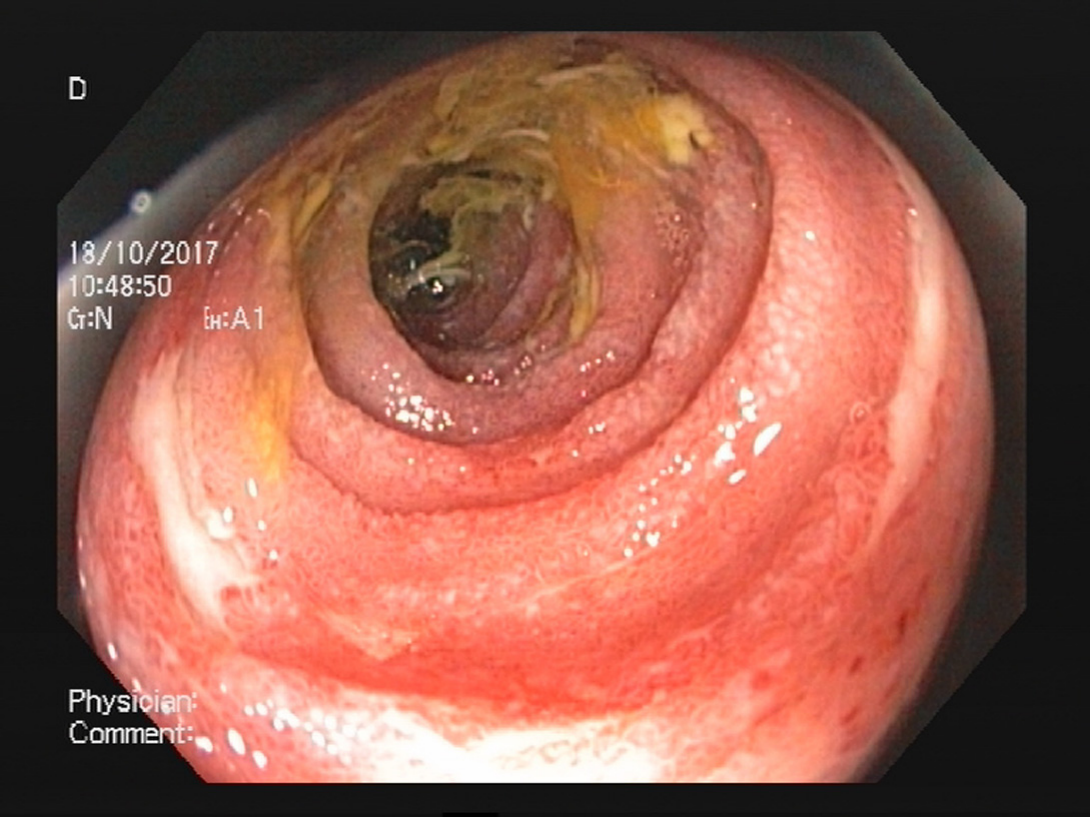
Colonoscopy demonstrating sections of ischemia.

**Figure 2:**
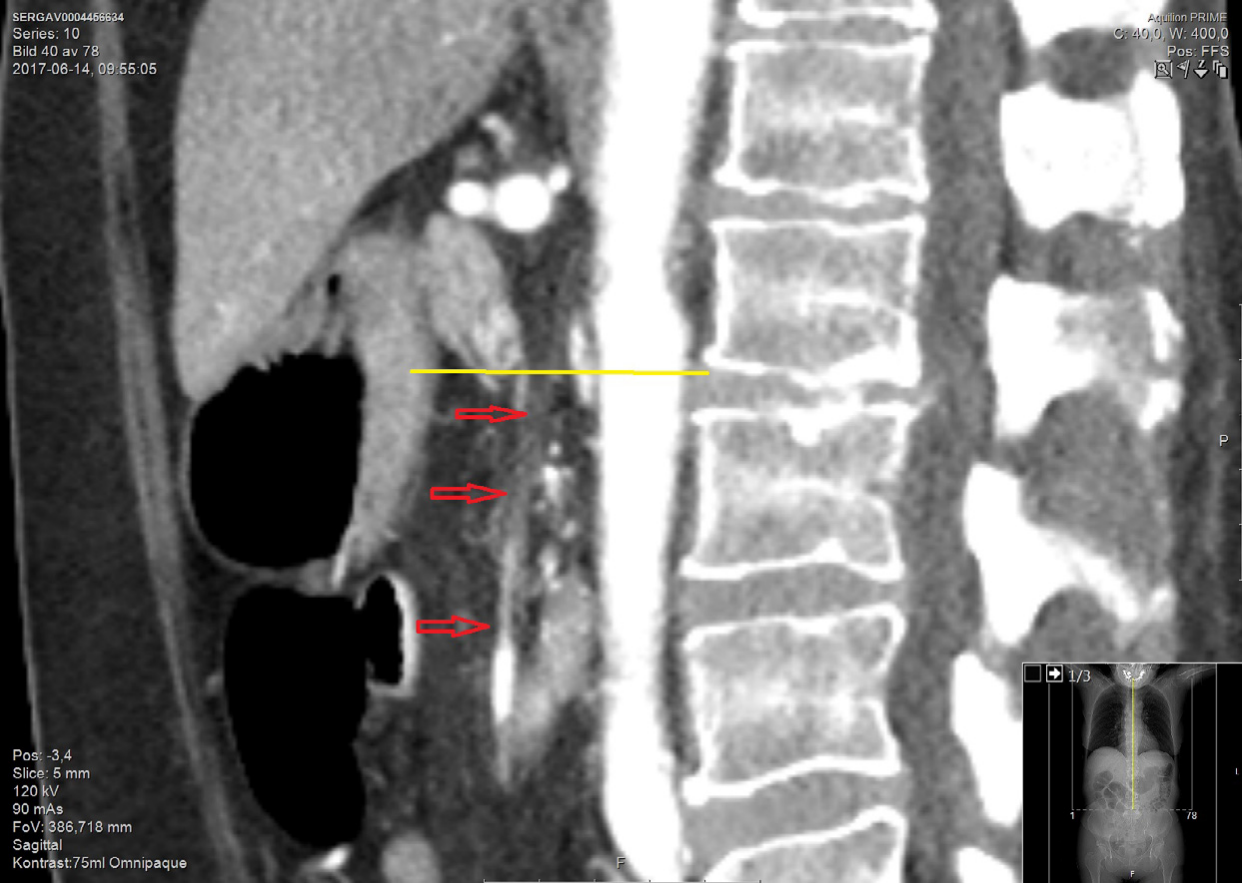
Abdominal computed tomography scan demonstrating the total occlusion of superior mesenteric artery (sagittal).

**Figure 3:**
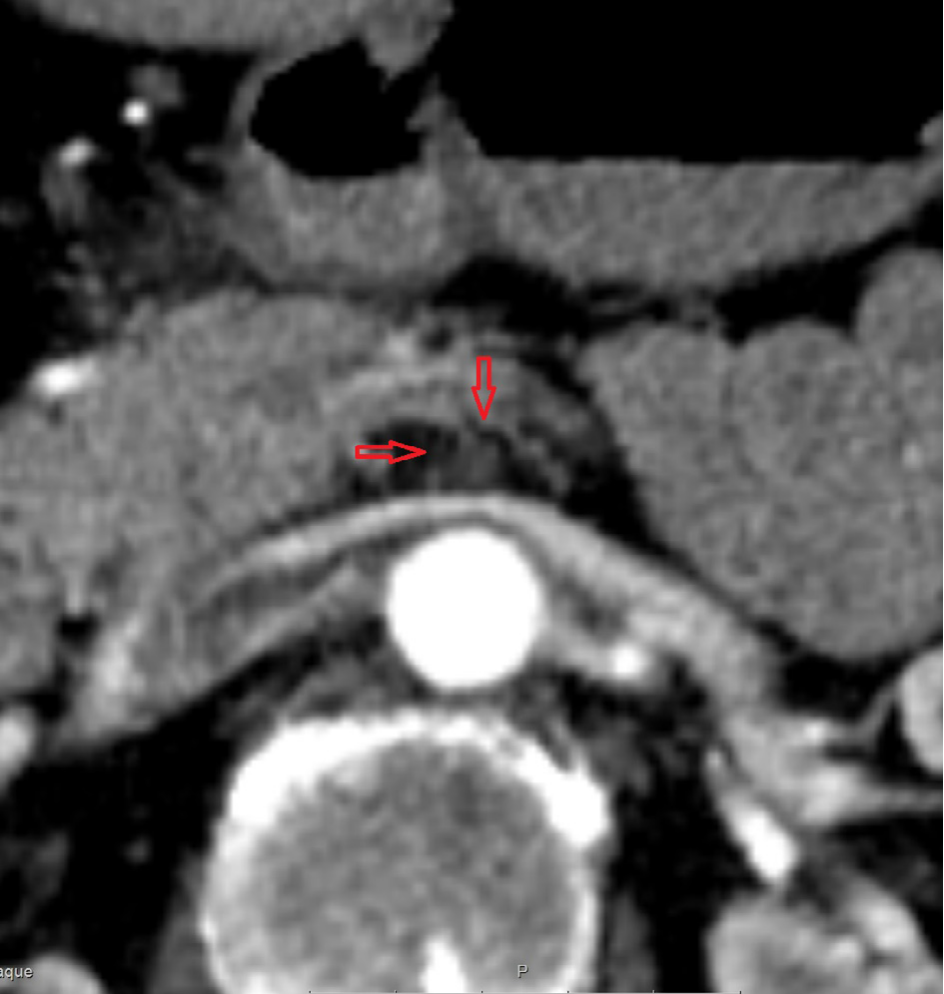
Abdominal computed tomography scan demonstrating the total occlusion of superior mesenteric artery (transversal).

**Figure 4:**
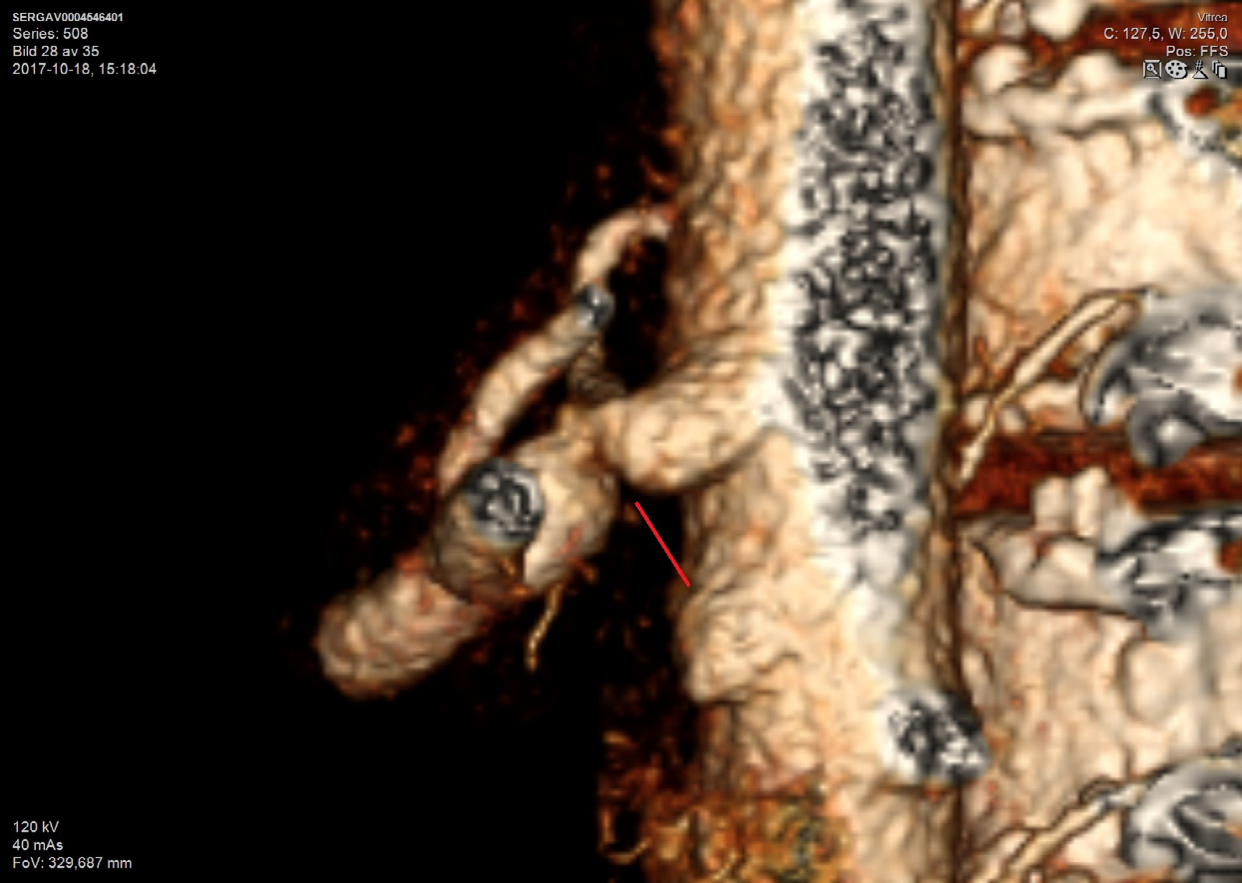
Abdominal computed tomography angiography showing the total occlusion of truncus coeliacus.

This leads to the suspicion of malignancy but CT of thorax and abdomen was normal in this regard. However, signs of hypoperfusion of the left kidney were revealed but the referring physician did not evaluate this further. Two years after the first onset of symptoms, she was referred to gastroenterology expert management. She had nausea, vomited and reported weight loss of 46 kg, and was immediately transferred from the outpatient clinic to the ward for parental nutrition and further evaluation. At this time, another colonoscopy revealed areas of ischemia of the terminal ileum (Fig. 1), later confirmed by biopsy. The CT abdomen that was performed prior to the patient's referral to the gastroenterology unit was now reviewed again by a third radiologist who discovered a 6 cm total occlusion of the superior mesenteric artery (SMA), with open truncus coeliacus (TC), and inferior mesenteric artery (IMA) (Figs 2 and 3). Subsequently, the patient underwent a CT angiography of the abdomen that disclosed not only the SMA occlusion but also a short severe stenosis of TC (Fig. 4), bilateral stenoses of the renal arteries, and a normal IMA. Magnetic resonance imaging showed a 6‐cm section of the distal ileum with wall thickening and impaired peristalsis compared to the other sections of the small intestine.

There was no history of atrial fibrillation, a 12‐lead electrocardiogram normal and no arrhythmias during 48 h of ambulatory monitoring. Transthoracic echocardiography confirmed a structural normal heart without thrombi and shunts.

Laboratory tests revealed a leukocyte count of 10,400/mm^3^, hemoglobin 10.9 g/dL, hematocrit 32%, and platelets 279,000/mm^3^. Coagulation tests showed an isolated prolonged activated partial thromboplastin time that did not normalize after mixing, indicating the presence of an inhibitor. Protein C, protein S, and antithrombin III levels were within normal ranges. Creatinine was normal. Notably, immunologic markers were elevated: IgM anticardiolipin antibodies titer of 158 kMPL/L (normal <40 kMPL/L), IgM 2‐glycoprotein I antibodies at 48 kE/L (normal <7 kE/L), and lupus anticoagulant. A repeated test after 4 months showed anticardiolipin antibodies 86 kMPL/L and IgM 2‐glycoprotein I antibodies at 60 kE/L, respectively. Further evaluation ruled out immunologic deficiency as a secondary cause. The patient was diagnosed as having APS associated with occlusions of the TC, the SMA, and bilateral stenoses of renal arteries. She was anticoagulated with low molecular weight heparin and switched to warfarin after abdominal surgical exploration. First, she underwent an explorative laparotomy that revealed an ischemic, but not yet gangrenous, small intestine. The occluded TC and SMA were both stented after angioplasty during an endovascular procedure. A second explorative laparotomy showed an intestinal perforation and areas of ischemia that necessitated the removal of two segments (15 cm each) of the small intestine, at 20 cm and 40 cm from the ileocecal valve.

Following interventions, she gained appetite, the previous postprandial pain disappeared and she gained 13 kg of weight during the next 6 weeks.

## Discussion

Mesenteric ischemia has an annual incidence of 5.4/100,000 and a majority are females (70%) 6, 7, 8. There are two forms of the disease, chronic MI that is also called abdominal angina and acute MI that is caused by arterial embolism, arterial thrombosis, mesenteric venous thrombosis, or nonocclusive MI, which are summarized in Table 1 9, 10. Progressive atherosclerotic disease occurs in more than 90% of cases of chronic MI 11. Patients with acute MI may have either moderate abdominal symptoms, which might lead to consideration of other diagnoses, or have severe abdominal pain 12. Contrariwise, chronic MI presents with more typical symptoms such as postprandial abdominal pain (typically 30–60 min after eating), early satiety, diarrhea or constipation (or both), nausea or vomiting (or both), fear of eating, and subsequent weight loss 13. While postprandial pain is associated with other abdominal diseases, including gastric reflux, peptic ulcer disease, biliary disease, pancreatitis, diverticular disease, inflammatory bowel disease, and gastroparesis, the predominant symptom for chronic MI is weight loss 8.

**Table 1:** Clinical features of mesenteric ischemia

Cause (%)	Presentation	Risk factors
Arterial embolism (50%)	Acute	Myocardial infarction, arrhythmia, rheumatic valve disease, endocarditis, cardiomyopathies, history of embolic events, ventricular aneurysms, recent angiography
Arterial thrombosis (20%)	Insidious onset with progression to constant pain	Atherosclerosis, prolonged hypotension, hypercoagulability, estrogen, diabetes mellitus
Nonocclusive (20%)	Acute or subacute	Hypotension, hypovolemia, low cardiac output, alpha‐adrenergic agonists, digoxin, beta‐blockers
Venous thrombosis (10%)	Subacute	Right‐sided heart failure, previous deep venous thrombosis, hepatosplenomegaly, primary clotting disorder, malignancy, hepatitis, pancreatitis, recent abdominal surgery or infection, estrogen, polycythemia, sickle cell disease

The diagnosis of APS is based on the presence of at least two criteria, one clinical and one laboratory, according to the Sapporo statement, and vascular thrombosis or fetal loss/premature birth is the hallmark 14. The annual incidence of APS is estimated to be around 5/100,000 and the prevalence approximately 40–50/100,000 15. Bowel involvement is the second most frequent manifestation of APS 16. Our patient had multiple vascular thromboses and fulfilled laboratory criteria (presence of IgM anticardiolipin antibodies >40 kMPL/L, IgM 2‐glycoprotein antibodies >40 kE/L, and lupus anticoagulant) were all positive at baseline and follow‐up at 4 months. Although it is standard care that patients with APS diagnosed with venous thromboembolism should receive oral anticoagulants to a target international normalized ratio (INR) of 2.0–3.0, controversy remains as to whether patients with APS diagnosed with arterial thrombosis should receive oral anticoagulants with a target INR of 2.0–3.0 or combined therapy with including antiplatelet therapy 17. Our patient received acetylsalicylic acid 75 mg and warfarin. In addition, she was prescribed atorvastatin 40 mg.

Despite the association between arterial thrombosis in APS and visceral ischemia, our literature search yielded 12 articles 4, 18, 19, 20, 21, 22, 23, 24, 25, 26, 27, 28.

Our patient represents a case of a chronic MI with acute thromboses in two arteries (SMA and TC), in addition to bilateral renal artery stenosis in APS. Although there were typical symptoms of chronic MI, with substantial weight loss initially and extreme at the end, she was misdiagnosed for an extended period of time. In addition, the fact that the two‐first radiologists who examined the first CT abdomen failed to detect the SMA thrombosis led to an even longer diagnostic delay that had devastating consequences for our patient. Finally, she underwent successful dilatation and stenting of the culprit vessels but required surgical removal of two segments of the small intestine. Fortunately, she gained weight after successful treatment, and her overall general health improved considerably. This case report underscores the importance of recognizing MI and APS as a rare cause of weight loss and epigastric pain related to food intake.

## Conclusions

The combined mesenteric ischemia and antiphospholipid syndrome is a rare combination but life‐threatening condition. It may be suspected in patients with postprandial pain, diarrhea, and weight loss. Careful evaluation of colonoscopy imaging techniques is crucial, and treatments nutrition, stenting, and anticoagulation improve outcome.

## Authorship

NM: gave the idea. AHY, NM, and RL: involved in patient management. AHY and RL: made a critical review of the manuscript. PM and NM: wrote the manuscript and involved in project management.

## Conflict of Interest

None declared.
